# Fault Diagnosis of Lithium Battery Modules via Symmetrized Dot Pattern and Convolutional Neural Networks

**DOI:** 10.3390/s25010094

**Published:** 2024-12-27

**Authors:** Meng-Hui Wang, Jing-Xuan Hong, Shiue-Der Lu

**Affiliations:** Department of Electrical Engineering, National Chin-Yi University of Technology, Taichung 411, Taiwan; wangmh@ncut.edu.tw (M.-H.W.); h949708804@gmail.com (J.-X.H.)

**Keywords:** convolutional neural network, lithium battery, symmetrized dot pattern, fault diagnosis

## Abstract

This paper proposes a hybrid algorithm combining the symmetrized dot pattern (SDP) method and a convolutional neural network (CNN) for fault detection in lithium battery modules. The study focuses on four fault types: overcharge, over-discharge, aging, and leakage caused by manual perforation. An 80.5 kHz high-frequency square wave signal is input into the battery module and recorded using a high-speed data acquisition card. The signal is processed by the SDP method to generate characteristic images for fault diagnosis. Finally, a deep learning algorithm is used to evaluate the state of the lithium battery. A total of 3000 samples were collected, with 400 samples used for training and 200 for testing for each fault type, achieving an overall identification accuracy of 99.9%, demonstrating the effectiveness of the proposed method.

## 1. Introduction

Lithium-ion batteries have become a crucial part of modern energy infrastructure, widely used in portable electronic devices, electric vehicles, and renewable energy storage systems. They have significantly impacted our work patterns, daily habits, and even contributed to sustainable development. As a result, ensuring the safe use of lithium-ion batteries has become a major challenge. Common lithium-ion battery faults include overcharging and over-discharging [1], internal short circuits [2], aging [3], manufacturing defects [4], and mechanical damage [5]. Early detection of battery module faults is vital for enhancing the stability and safety of energy storage systems. The academic community has extensively studied battery fault diagnosis technologies, which can be broadly categorized into model-based methods and data-driven methods. Model-based diagnostic approaches, such as equivalent circuit models [6], electrochemical models [7], and thermal models [8], are typically used to analyze and predict battery health. However, these methods involve complex mathematical derivations and rely on precise parameter estimation, limiting their practical application. While model-based methods offer physical interpretability and accuracy, they come with higher adaptation costs and development complexity. In contrast, data-driven methods such as machine learning [9], deep learning [10], and data analysis [11] can automatically learn fault patterns from historical data, reducing reliance on complex physical models and proving more suitable for real-world applications. With the successful application of deep learning in image recognition, speech recognition, and semantic analysis [12,13], its potential in intelligent fault diagnosis has garnered widespread attention. For example, Reza Rouhi Ardeshiri et al. [14] proposed a method to convert battery signals into multi-channel images, and used a 2D convolutional neural network (CNN) to automatically extract features from these images to achieve an online estimation of the state of charge (SOC) of the battery. Ming Xing You et al. [15] introduced a health assessment method based on voltage, current, and temperature data extracted during battery charging and discharging processes. These data were divided into training, validation, and testing sets, and a long short-term memory (LSTM) recurrent neural network was used to develop a battery capacity estimation model for evaluating the state of health (SOH) of the battery. Zhi Wei Dong et al. [16] compared the performance of backpropagation neural networks, LSTM, and CNNs, finding that CNNs excelled in estimating the SOC of low-charge batteries. Hang Yuan et al. [17] proposed a fault diagnosis method combining Symmetrized Dot Pattern (SDP) and CNNs, which calibrated current signals through phase adjustment to improve the robustness of the neural network. They considered factors affecting the current signal, computed the calibration residual signal (CRS), and amplified subtle fault information in the current signal. These CRSs were converted into SDP images with color information and used for fault pattern recognition through a pre-trained 2D CNN.

Building on the application of deep learning in lithium battery fault diagnosis as presented in the above literature, this study proposes a fault recognition method combining Symmetrized Dot Pattern (SDP) [18] with convolutional neural networks (CNNs) [19] to detect four types of lithium battery module faults. In the experiments, we used the NI PXI-5105 to collect battery signals, which were then converted and processed into SDP input images. These images were subsequently analyzed using CNNs to extract features and identify faults. Additionally, we compared this method with advanced deep learning models such as ResNet [20] and VGG [21] to evaluate recognition accuracy. CNN, as a fundamental deep learning model, performs excellently in image classification tasks and is a natural choice for this study. Meanwhile, VGG and ResNet, as more complex advanced network architectures, demonstrate outstanding performance in image recognition tasks. By comparing these models, we aim to assess the differences in recognition accuracy between simpler and more complex models in fault diagnosis, providing a more comprehensive perspective on the application of deep learning in lithium battery fault recognition. Experimental results show that this method effectively identifies lithium battery fault types with high recognition accuracy.

## 2. Methodology

### 2.1. Symmetric Data Point Coordinate Method

**Figure 1:**
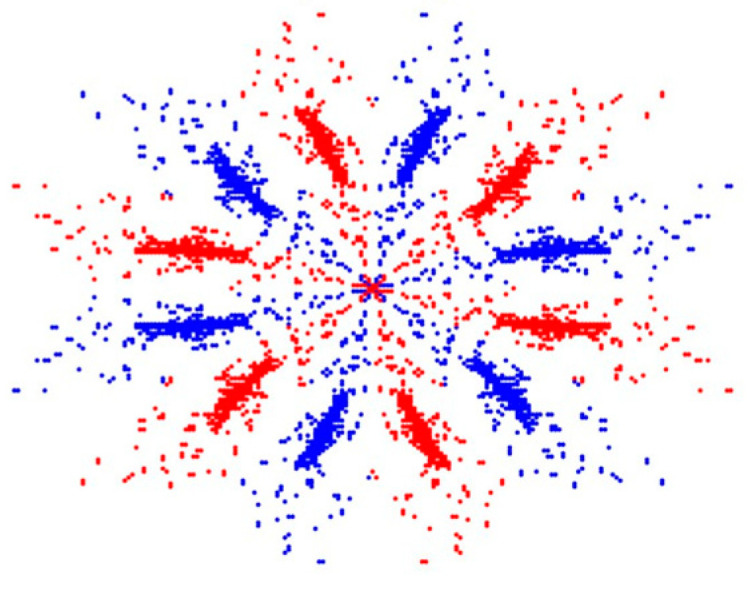
The snowflake pattern of the lithium battery under normal conditions.

This study proposes a fault detection method for lithium battery modules based on the Symmetrized Dot Pattern (SDP) coordinate system. The SDP algorithm transforms the raw signals of faulty modules into two-dimensional, snowflake-like patterns, capturing the characteristics of lithium batteries under different fault conditions as key features for fault identification. An examples of these snowflake patterns is shown in Figure 1.

Using the SDP method, the voltage signals are converted into two-dimensional images, where the X-axis and Y-axis correspond to the horizontal and vertical pixel coordinates of the image, respectively. These images reflect texture features associated with different fault types. The images are then cropped into 64 × 64 pixel feature maps, which serve as an input to train a convolutional neural network (CNN) for feature extraction and fault classification.

**Figure 2:**
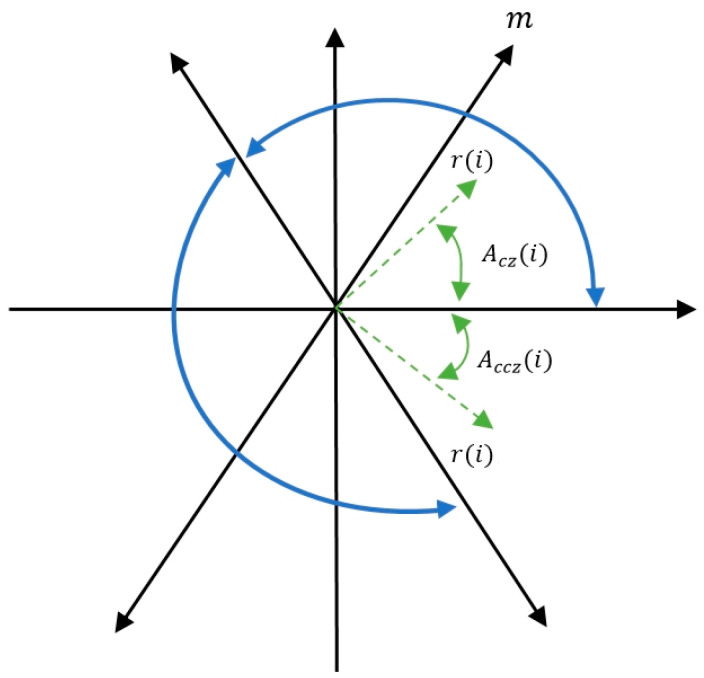
Symmetrized dot pattern.

The symmetrized dot pattern (SDP) method [22] is a data transformation technique used to process time-domain square wave signals. This method involves converting the signal into polar coordinate feature images through a data extraction system. Figure 2 demonstrates the principle of this method, where the radius (*i*), the clockwise rotation angle $A_{cz}$(i), and the counterclockwise rotation angle $A_{ccz}$(i) are the three main features of the image. In the sampled square wave data of the lithium battery module state signal, each signal sampling point $Z_{i}$ represents the signal strength at time i, while the signal sampling point $Z_{i+\tau }$ represents the signal strength at time i+τ. By substituting these data into Equations (1)–(3), the transformed polar coordinate points O(*r*(*i*), $A_{cz}(i)$, $A_{ccz}$(i)) can be obtained. By changing the initial rotation angle, different coordinate symmetric images of the lithium battery module state signal can be generated. In summary, the symmetrized dot pattern method is an effective signal processing technique that aids in the analysis and understanding of the state of lithium batteries.

(1) $$r(i)=\frac{Z_{i}-x_{min}}{x_{max}-x_{min}}$$

(2) $$A_{cz}(i)=\varphi -\frac{Z_{i+\tau }-x_{min}}{x_{max}-x_{min}}Q$$

(3) $$A_{ccz}(i)=\varphi +\frac{Z_{i+\tau }-x_{min}}{x_{max}-x_{min}}Q$$

The feature map parameters in this study are designed to analyze and identify faults in lithium battery modules. These parameters are defined as follows: $x_{max}$ represents the maximum value of the original signal, $x_{min}$ represents the minimum value of the original signal, τ denotes the time interval parameter between signals, (1 ≤ τ ≤ 10) represents the initial offset angle on the x-axis, and (φ) is the amplification coefficient for the rotation angle (Q≤θ). Experimental tests found that setting φ to 60°, τ to 3, and Q to 3 effectively aids in identifying and analyzing faults in lithium battery modules.

### 2.2. Convolutional Neural Network

Convolutional neural networks (CNNs) originated in the late 1980s to early 1990s [23], proposed by French computer scientist Yann LeCun and his team. In 1989, LeCun and others developed the LeNet model, an early prototype of CNNs. LeNet-5 demonstrated the potential of CNNs in image data processing, laying the foundation for more complex CNN architectures. Since then, CNNs have been widely applied in fields such as image recognition [24], medical image analysis [25], and fault diagnosis [26]. CNNs have not only significantly reduced manual labor and time consumption but have also driven the extensive application of deep learning in computer vision, facilitating technological progress in advanced deep convolutional networks.

#### 2.2.1. Convolutional Kernel and Convolution Layer

**Figure 3:**
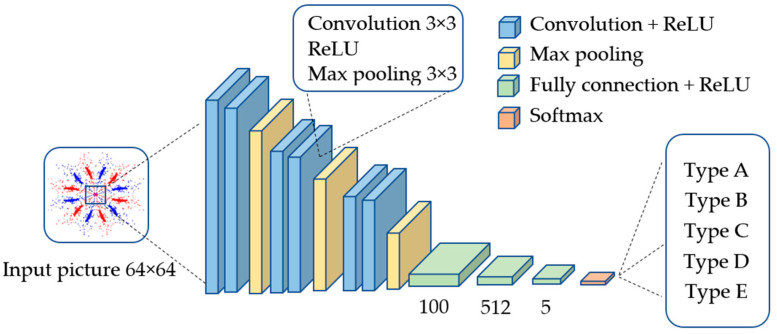
Convolutional neural network architecture.

The CNN architecture employed in this study features three convolutional layers and three fully connected layers. Each convolutional layer utilizes a 3 × 3 kernel paired with the ReLU activation function. The first convolutional layer focuses on extracting fundamental features such as edges, lines, and textures. The second layer builds on this by identifying more complex patterns like shapes and angles. The third layer further refines the process, detecting high-level features that enable the model to recognize intricate patterns within the images. The detailed structure of the CNN model used in this study is illustrated in Figure 3.

The model then processes information through three fully connected layers, outputting the final classification. The first layer outputs 1000 neurons, the second layer outputs 512 neurons, and the final layer outputs 5 neurons. This design effectively handles image classification tasks, using the ReLU activation function and a softmax layer to ensure classification accuracy.

In the CNN model, the convolutional kernel [27] is an essential component of the convolutional layer. The calculation for the feature map size after the convolution operation is shown in Equation (4). ${FZ}_{out}$ represents the size of the output feature map, ${FZ}_{in}$ is the size of the input feature map, f denotes the size of the convolutional kernel, Stride is the step size of the kernel’s movement for each convolution, and P is the zero-padding value.

(4) $${FZ}_{out}=\frac{{FZ}_{in}-f+2P}{Stride}+1$$

#### 2.2.2. Pooling Layer

The pooling layer in CNNs reduces the size of feature maps through downsampling, lowering computational and memory requirements while retaining key features. Common pooling methods include max pooling and average pooling. Max pooling selects the maximum value in a region, emphasizing features like edges and textures, while average pooling averages values in a region, smoothing the feature map. The alternating use of convolutional and pooling layers reduces feature map resolution and extracts abstract features, which is beneficial for complex image processing tasks.

#### 2.2.3. Fully Connected Layer

**Figure 4:**
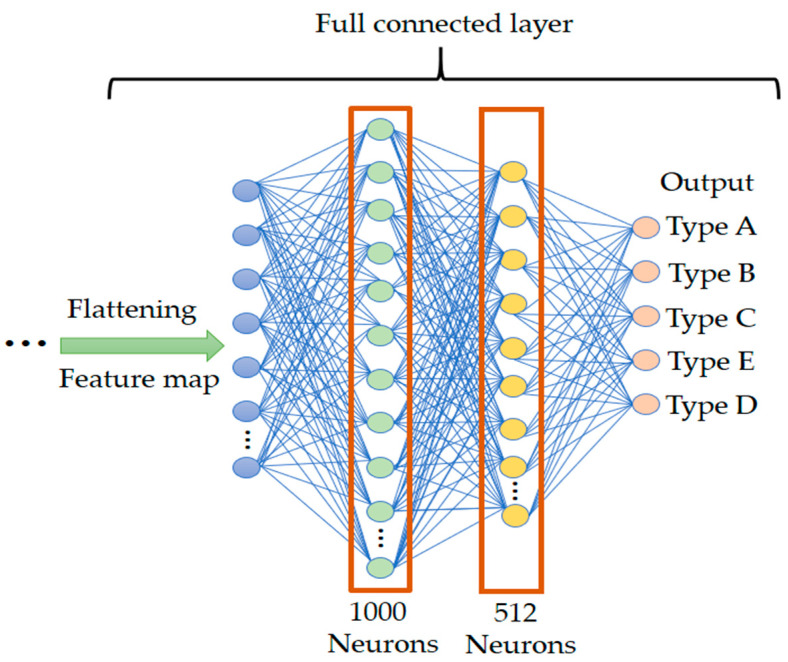
Convolutional neural network fully connected layer architecture.

The fully connected layers in this study, as shown in Figure 4, are placed after the convolutional layers and handle the final classification or prediction. The model has three fully connected layers: the first layer with 1000 neurons, the second with 512 neurons, and the last with 5 neurons for classification output. These layers integrate features from the convolutional layers into a feature vector, then use the softmax function to calculate the class probability distribution for classification.

The softmax function [27] is commonly used in multi-class classification problems. It transforms a K-dimensional real-valued vector into a K-dimensional probability distribution, ensuring each element is between 0 and 1, and the sum of all elements equals 1. In practical applications, this allows us to identify the class with the highest predicted probability. The function is commonly used in multi-class classification tasks to convert the model’s output into probability values. Its mathematical formula is shown in Equation (5). Take the vector Z, where $Z_{S}$ denotes the s-th element of the vector Z, and n is the number of elements in Z. The output of the softmax function, $z_{s}$, represents the probability distribution generated by the softmax function.

(5) $$softmax\;\left(z_{s}\right)=\frac{\exp\;\left(z_{s}\right)}{\sum_{s=0}^{n}\exp\;\left(z_{s}\right)}\;where\;z=0,\ldots ,n$$

## 3. Research System Architecture and Fault Design

### 3.1. Experimental System Process Architecture

**Figure 5:**
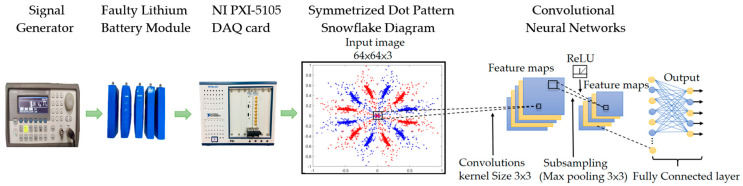
Architecture of system.

This study detects fault signals in lithium battery modules using an experimental platform. A waveform generator inputs a high-frequency square wave signal into the module. The load signal is captured by a high-speed data acquisition card, processed into a snowflake pattern using the symmetrized dot pattern (SDP) method, and fault types are identified using a CNN. The system architecture is shown in Figure 5.

**Figure 6:**
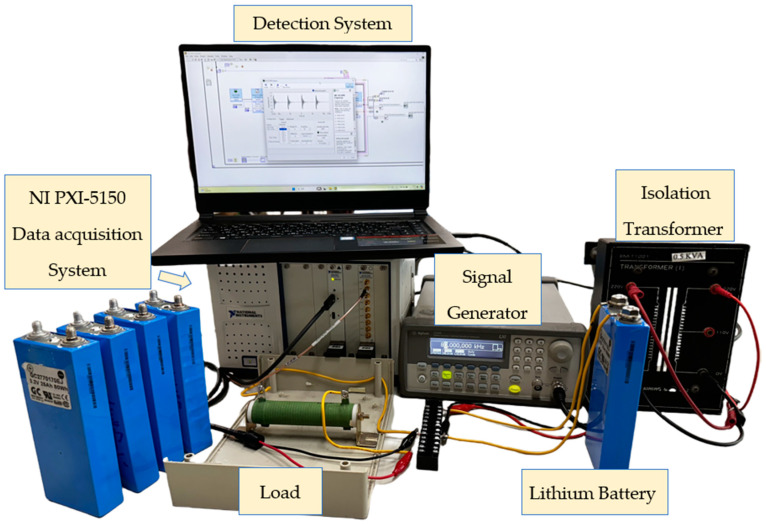
Lithium battery model testing platform.

Figure 6 shows the experimental platform combining the NI PXI-5105 data acquisition card and the NI PXIe-1071 chassis for fault signal detection. This system features a 60 MHz sampling frequency, 8 synchronized channels, and 12-bit resolution, with built-in memory and instrument drivers for data streaming and analysis, allowing comprehensive capture of lithium battery fault signals.

### 3.2. Design of Experimental Fault Models for Lithium

This experiment established five lithium battery fault signal models, including: Model A for normal state, Model B for over-discharge, Model C for overcharge, Model E for aging, and Model D for simulating leakage caused by external forces. Detailed information about the models is summarized in Table 1. The lithium battery used in the experiment has the following specifications: a nominal voltage of 3.2 V, a capacity of 25 Ah, a weight of 600 g, a cycle life of 2500 cycles, a charging voltage of 3.65 V, a charging current of 17.5 A, a discharge voltage of 2.5 V, and a discharge current of 25 A.

**Figure 7:**
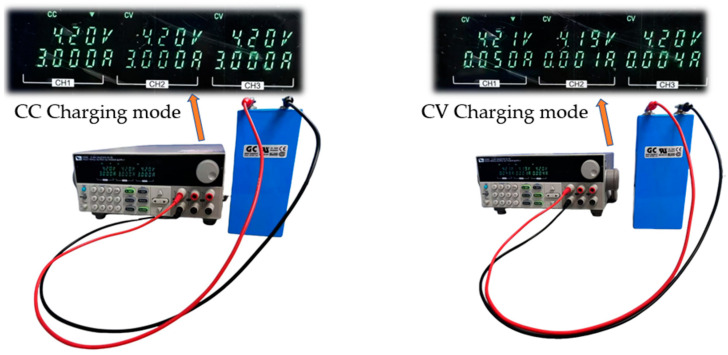
Lithium battery pre-conditioning experiment.

In this study, a 3.2 V, 25 Ah lithium iron phosphate (LiFePO4) battery was used for the experiments. All the batteries were preconditioned, as shown in Figure 7, to ensure consistent charge levels. Initially, each battery was charged to 4.2 V using the constant current mode (0.12 C, 3 A), and then switched to the constant voltage mode until the current dropped below 0.05 A. The experimental setup also included various equipment to manage and monitor the charging and discharging processes, which are summarized in Table 2.

#### 3.2.1. Normal (Type 1)

**Figure 8:**
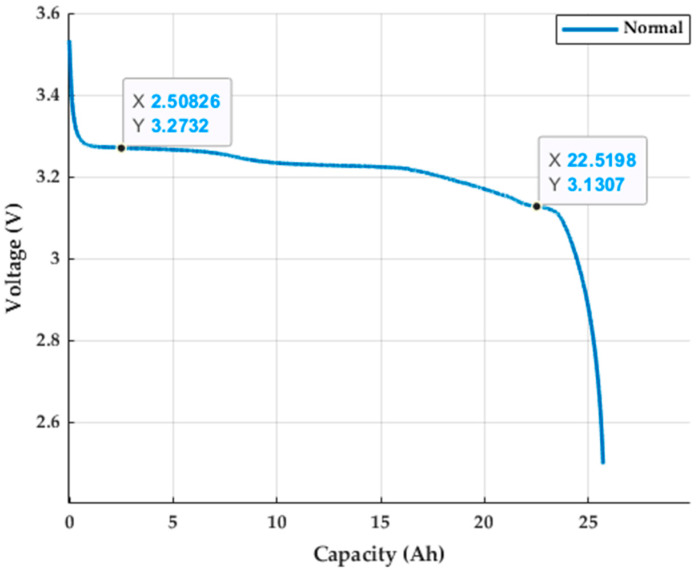
Lithium battery discharge voltage capacity curve.

**Figure 9:**
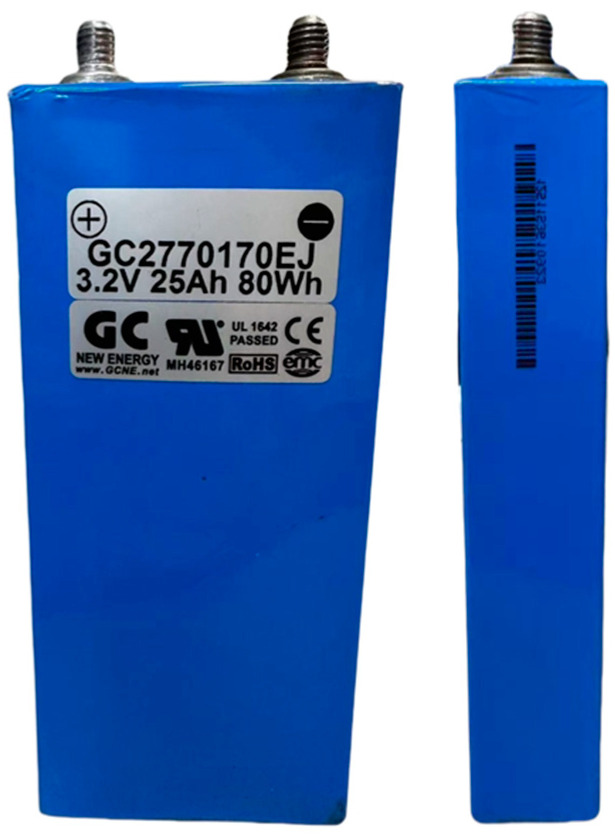
Normal appearance of the lithium battery.

Lithium batteries, as core components in energy storage systems, play a critical role in energy storage and release. Under normal operating conditions, lithium batteries should exhibit stable voltage and capacity, consistently provide the designed output power, and maintain a long service life within the specified charge and discharge cycles. The lithium iron phosphate (LiFePO4) battery used in this study has a performance specification of 3.2 V, 25 Ah, and 80 Wh, which meets its technical requirements. The test was conducted using a programmable electronic load for a 3 A (0.12 C) discharge test, as shown in Figure 8. The measured discharge voltage and capacity curves were obtained at an ambient temperature of 24 °C, with the operating voltage ranging from 3.13 V to 3.27 V, in accordance with the manufacturer’s standard for LiFePO4 batteries. Figure 9 shows the appearance of the normal lithium battery used in this study.

#### 3.2.2. Over-Discharging (Type 2)

When lithium batteries are over-discharged beyond their design limits, it results in excessively low internal voltage, causing permanent capacity loss. According to the literature [28], low-voltage conditions pose risks such as irreversible electrode damage, reduced charging efficiency, and shorter cycle life, potentially leading to irreversible damage.

**Figure 10:**
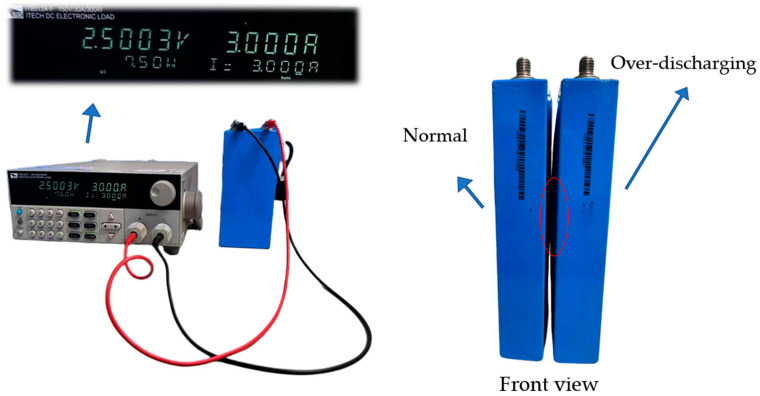
Lithium battery over-discharge experiment.

In this study, after preheating a single lithium battery model, discharge was performed at 3 A with a cutoff voltage of 2.5 V, followed by a 30 min cooling period. The battery was then charged at 3 A in the constant current (CC) mode until it reached 4.2 V, at which point the charging mode switched to constant voltage (CV) until the current dropped to 0.05 A. A subsequent 30 min cooling period followed. This cycle was repeated 10 times. As shown in Figure 10, the voltage dropped to 2.5 V, and the over-discharged battery model exhibited a slight swelling at the center compared to a normal battery.

#### 3.2.3. Overcharging (Type 3)

Long-term overcharging can cause an imbalance in the chemical reactions inside a lithium battery, leading to swelling, temperature rise, and performance degradation. According to reference [29], the risks of overcharging a lithium battery in high-temperature environments include thermal runaway, reduced capacity, and shortened lifespan, which not only affect performance but also pose safety hazards.

**Figure 11:**
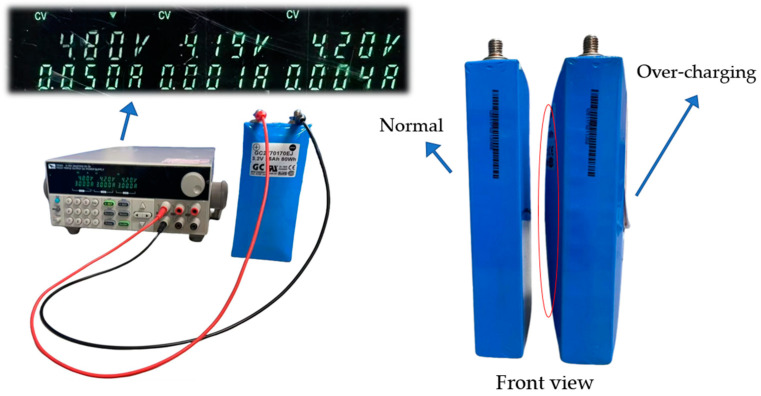
Lithium battery overcharge experiment.

In this study, the battery was preheated and charged at 3 A constant current until it reached 4.8 V, then switched to the constant voltage mode until the current dropped below 0.05 A. After charging, a 30 min cooling period was applied. As shown in Figure 11, the overcharged battery model displayed significant swelling in the area circled in red, compared to the normal battery model.

#### 3.2.4. Leakage (Type 4)

**Figure 12:**
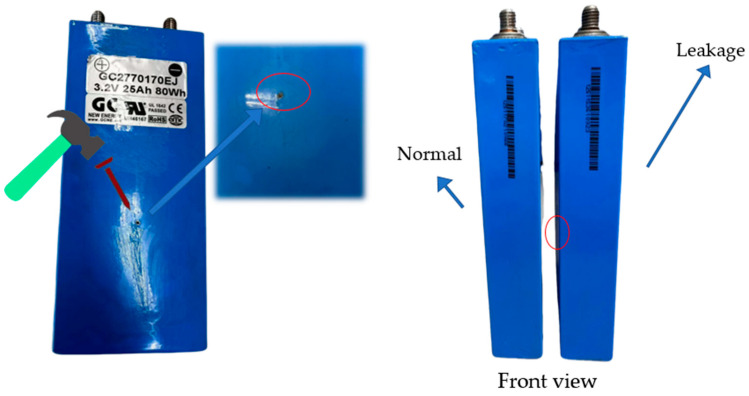
Lithium battery leakage experiment.

Battery leakage is typically caused by manufacturing defects, external impacts, overcharging, and packaging issues. Leakage can corrode the battery casing and internal components, potentially damaging the surrounding environment and equipment. It leads to performance degradation, increased internal resistance, and capacity loss, and in severe cases, can even cause fires [30]. In this study, a steel nail was used to puncture the battery model lightly, and the battery was left to stand for 20 days to simulate leakage. As shown in Figure 12, there are no visible differences in appearance, but the leakage area, circled in red, shows slight swelling compared to the normal battery model when touched.

#### 3.2.5. Aging (Type 5)

**Figure 13:**
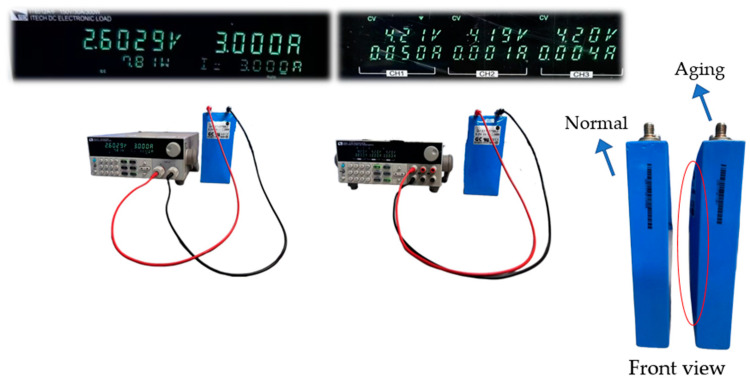
Lithium battery aging experiment.

The aging of lithium batteries is typically associated with factors such as manufacturing defects, charge–discharge cycles, high-temperature environments, overcharging, over-discharging, and external impacts. According to reference [31], after aging, the battery’s efficiency declines, its lifespan shortens, and the cutoff discharge voltage decreases, affecting the normal operation of the device. In this study, the battery was charged at 3 A under the constant current mode until it reached 4.2 V, then switched to the constant voltage mode until the charging current dropped to 0.05 A. After charging, a 10 min cooling period was applied, followed by discharging at 3 A with a cutoff voltage of 2.6 V to prevent over-discharging, and another 10 min cooling period. This charge–discharge cycle was repeated 20 times to simulate excessive use and battery aging. As shown in Figure 13, significant swelling was observed in the area circled in red on the battery’s surface.

### 3.3. Fault Model Capacity Experiment

**Figure 14:**
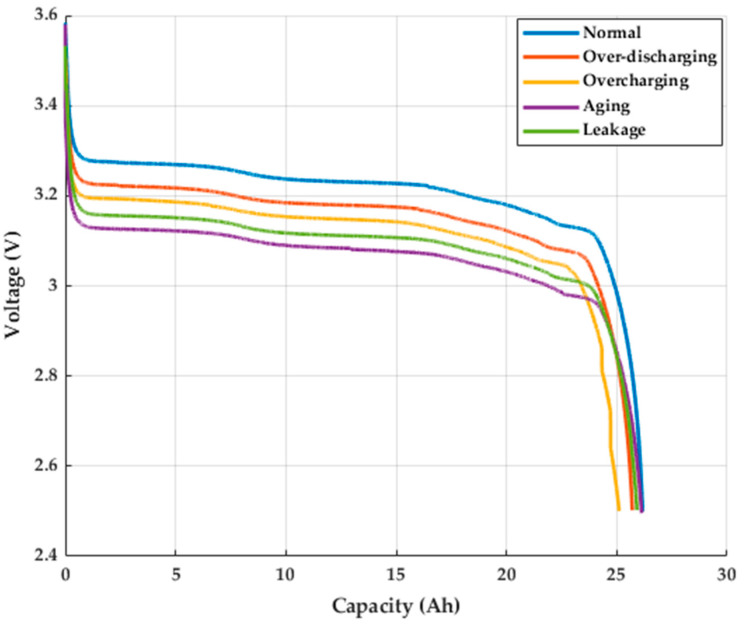
Lithium battery discharge capacity test.

In our study, after completing the creation of fault models and conducting capacity tests for the four fault types as shown in Figure 14, it was observed that the capacity of each fault model showed significant degradation compared to the normal model. The most significant capacity degradation was caused by aging, followed by leakage, over-discharge, and overcharge. Aging primarily causes capacity degradation due to the failure of internal chemical reactions and the gradual loss of electrode materials, leading to a reduction in the battery’s energy storage ability. The leakage model reduces battery performance by disrupting internal chemical reactions. Over-discharge causes the battery voltage to drop too low, damaging the battery structure and resulting in irreversible capacity loss. Overcharge causes the battery voltage to become too high, damaging the electrodes and electrolyte, leading to capacity degradation and increased safety risks.

## 4. Experimental Results

### 4.1. Original Waveform Measurement

Before starting the experiment, the lithium battery was charged using a constant current–constant voltage (CC-CV) mode, with a preset charging voltage of 3.5 V and a current threshold of I < 0.5 A. Signal acquisition was conducted for five fault types. A high-frequency square wave signal with a frequency of 80.5 kHz and 20 Vp-p was input. Data were sampled at 25 μs intervals over four cycles. A total of 3000 samples were collected, with 400 used for training and 200 for testing per fault type.

**Figure 15:**
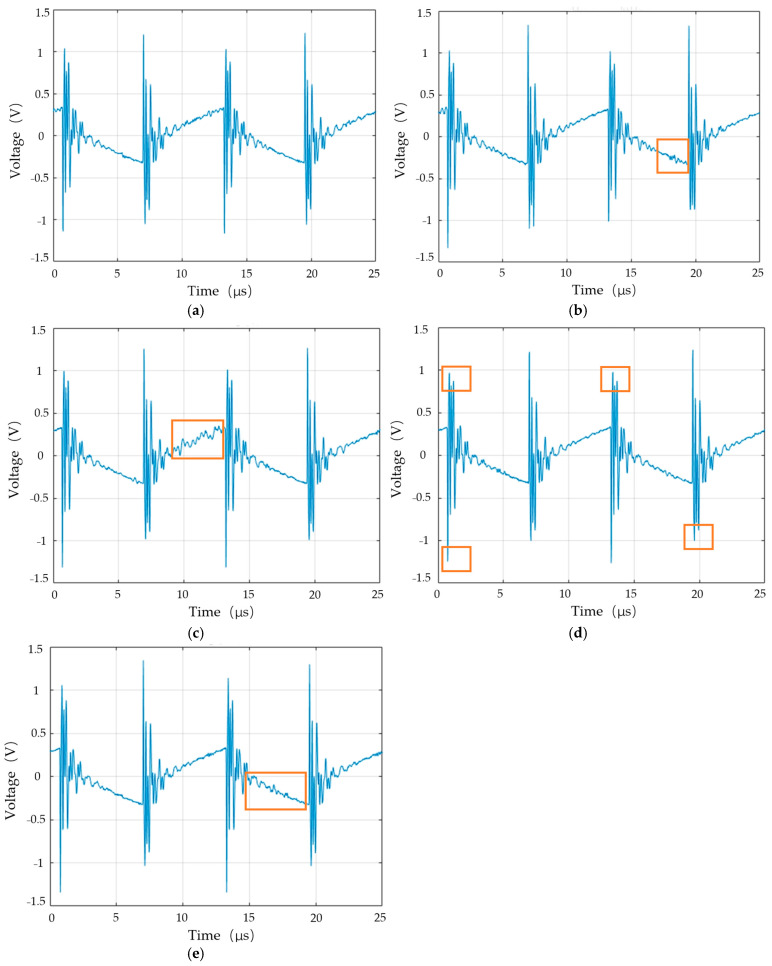
Signals of lithium battery fault types: (**a**) normal, (**b**) over-discharge, (**c**) overcharge, (**d**) leakage, and (**e**) aging.

As shown in Figure 15a, the output voltage waveform of the lithium battery is affected by the voltage drop across the load, resulting in a decrease in peak voltage. The main differences in the battery signals are observed in the voltage fluctuations within the positive and negative half-cycles. Therefore, voltage fluctuations associated with different fault types can serve as identification signals. In Figure 15b, comparing the over-discharge model with the normal model, a small oscillation is observed near the voltage drop in the 15 to 20 μ/s range. In Figure 15c, the overcharge model shows larger voltage fluctuations compared to the normal model during the 10 to 15 μ/s period. In Figure 15d, the leakage model exhibits differences from the normal model in terms of voltage peak fluctuations in the 0 to 5 μ/s range in both the positive and negative half-cycles, as well as a decrease in peak voltage in the 10 to 20 μ/s range. Finally, comparing the aging model in Figure 15e with the normal model, a slight oscillation around 15 to 20 μ/s is observed, similar to the oscillation seen in the over-discharge model in Figure 15b.

### 4.2. Application of the Symmetric Point Coordinate Method in Lithium Battery Modules

**Figure 16:**
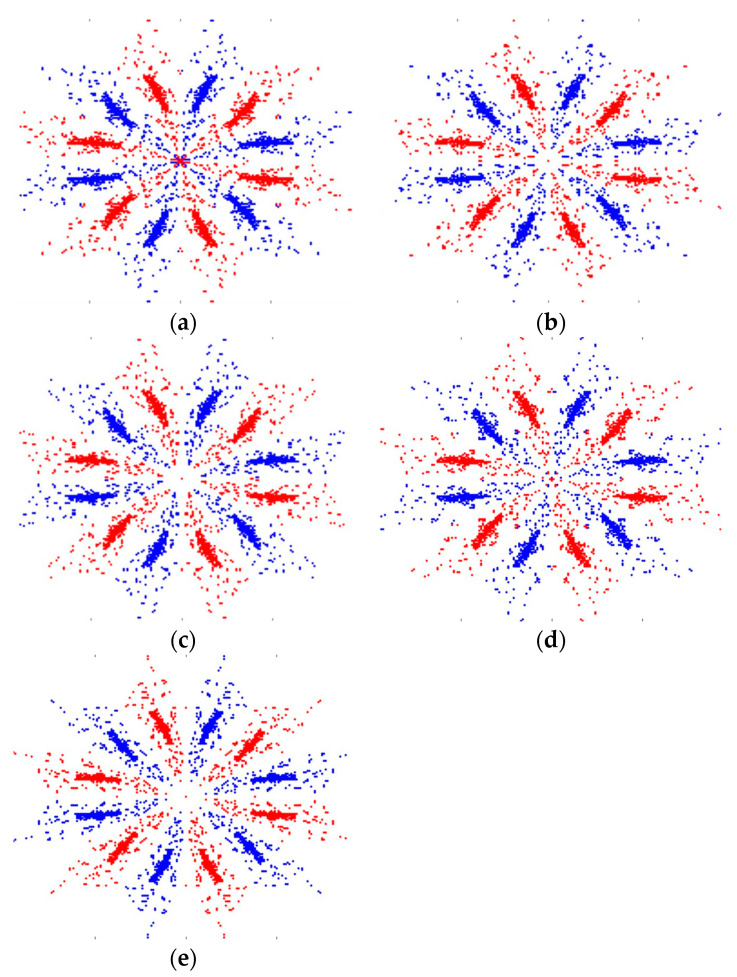
Snowflake diagram of lithium battery fault types: (**a**) normal, (**b**) over-discharge, (**c**) overcharge, (**d**) leakage, and (**e**) aging.

This study focuses on analyzing the waveform signals generated by lithium battery modules and inputting the data into the SDP system to produce corresponding feature maps. Figure 16 displays the feature images for the five types of lithium battery module faults, which are essential for fault detection and diagnosis.

The snowflake patterns of lithium batteries under different fault conditions have distinct features for fault identification. The distribution and density of these patterns vary with the fault type. Significant differences can be observed when comparing the fault model to the normal model, primarily concentrated at the center. This feature was applied in deep learning for fault training and identification.

### 4.3. Recognition Results of the CNN

In this study, the CNN model for fault type identification used 3000 snowflake feature images, with 400 training samples and 200 testing samples for each fault type. The method effectively classified lithium battery module faults, achieving a recognition accuracy of 99.9%, as shown in Table 3. The high accuracy is attributed to the CNN’s powerful deep feature extraction capability, while batch normalization improved training stability and addressed the vanishing gradient problem. The SGDM optimizer ensured fast convergence, and proper data partitioning enhanced the model’s generalization, resulting in excellent performance in the lithium battery image recognition task.

**Figure 17:**
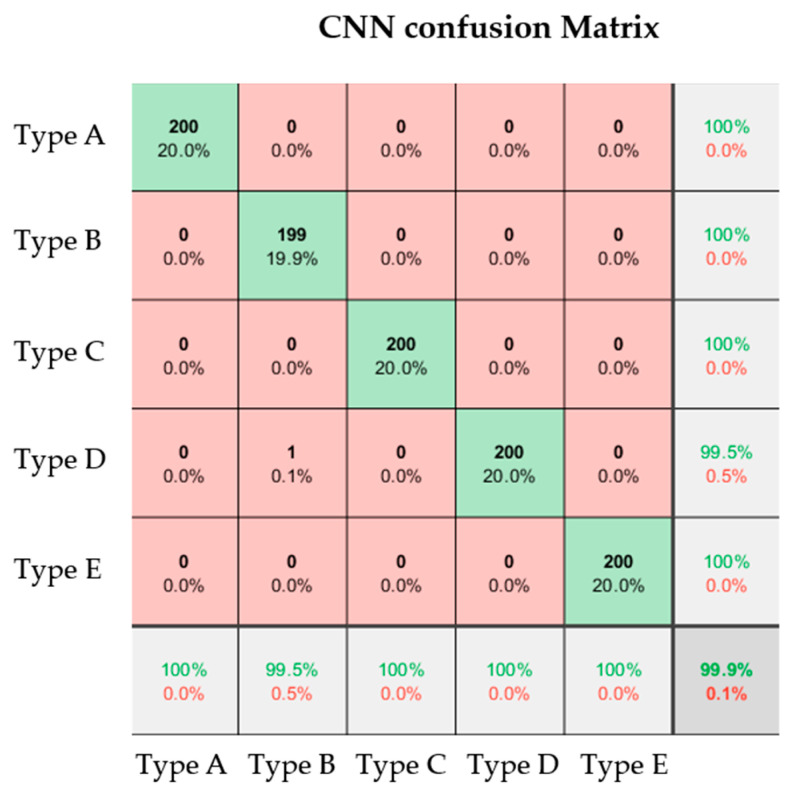
Confusion matrix for fault diagnosis of lithium battery modules based on convolutional neural networks.

Figure 17 shows the CNN confusion matrix, with the horizontal axis representing actual fault types and the vertical axis representing predicted fault types. Green indicates correct classifications, while red indicates misclassifications. The accuracy and misclassification rates for each fault type are shown in the light gray grid, and the overall accuracy and misclassification rates are in the dark gray grid at the bottom right.

The results show that the 200 Type A (normal battery) samples were classified with 100% accuracy. Type B (over-discharged battery) had an accuracy of 99.9%, with one sample misclassified as a leakage battery. Type C (overcharged battery), Type D (leakage battery), and Type E (aging battery) all showed excellent classification, with all 200 samples correctly classified.

**Figure 18:**
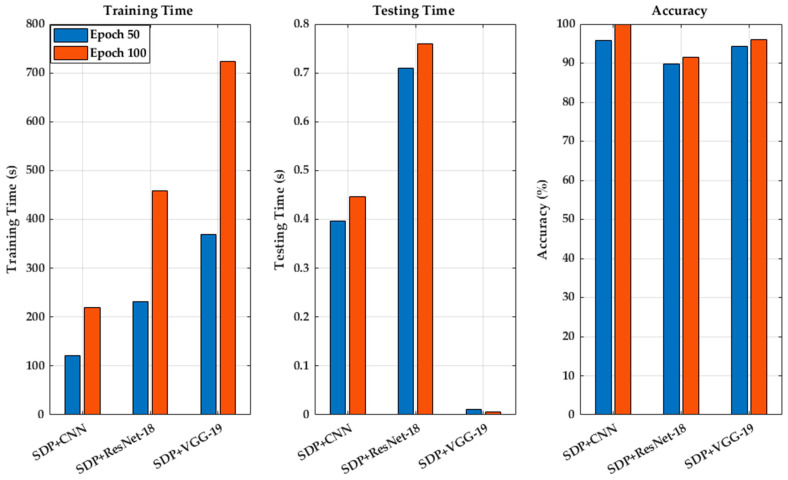
Performance differences among different algorithms.

This study demonstrates the high accuracy achieved by combining SDP and CNN, as shown in Figure 18 and Table 4. When the number of training epochs is set to 50, the accuracy is 95.7%, and it increases to 99.9% after 100 epochs, with the best results obtained at 100 epochs. The study also explores advanced deep learning algorithms, such as ResNet-18 and VGG-19. At 50 epochs, SDP + CNN achieved 95.7% accuracy in 121.9 s, while SDP + ResNet-18 achieved 89.8% accuracy in 232 s, and SDP + VGG-19 achieved 94.4% accuracy in 369.6 s. At 100 epochs, SDP + CNN reached 99.9% accuracy in 218.5 s, SDP + ResNet-18 achieved 91.5% accuracy in 458.6 s, and SDP + VGG-19 achieved 96.0% accuracy in 723.5 s. In conclusion, SDP + CNN performed excellently at both 50 and 100 epochs, in terms of both training time and accuracy. In comparison, although ResNet-18 and VGG-19 also showed high accuracy, their training times were significantly longer, especially VGG-19, which had a much longer training time than CNN. Ultimately, the combination of SDP and CNN achieved the best performance in lithium battery fault recognition, in terms of both accuracy and training time.

## 5. Discussion

This study combines the methods of snowflake diagrams and convolutional neural networks (CNN), particularly for their effectiveness in lithium battery fault diagnosis. Compared to model-based fault diagnosis methods, our approach offers significant advantages, as model-based methods typically rely on complex mathematical models and assumptions, while our research transforms battery voltage signals into images using snowflake diagrams and employs CNN for automatic feature extraction, avoiding extensive physical assumptions. This enables us to capture both temporal and spatial features of the signals, improving fault classification accuracy and offering stronger adaptability. However, the method may face challenges when dealing with more complex fault types, such as thermal runaway and internal short circuits, where model-based methods generally provide theoretical support. Our method, on the other hand, requires more training data to maintain high accuracy and still needs to improve robustness and generalization in multi-fault-type recognition. To further enhance fault diagnosis accuracy and robustness, we suggest that future research could involve experiments at different temperatures to explore the impact of temperature variations on battery fault diagnosis. Additionally, future studies should focus on including more fault types, particularly common fault modes in practical applications, such as thermal runaway and internal short circuits, to improve the practicality and performance of fault diagnosis models.

## 6. Conclusions

This paper proposes a method for lithium battery module fault diagnosis by combining snowflake patterns and convolutional neural networks (CNNs). By extracting the signal features of the battery under load conditions, the method can accurately determine the battery’s state. The study covers five different battery states: normal, overcharge, over-discharge, leakage, and aging. The experimental results show that as the number of training epochs increases from 50 to 100, the CNN model’s recognition accuracy improves significantly, achieving optimal performance. The study also compares advanced deep learning algorithms, such as ResNet and VGG, and the results show that CNN outperforms these two more complex network architectures in terms of fault recognition accuracy and training efficiency. While ResNet and VGG are more suitable for processing complex images, CNN performed better in this study. These results validate the effectiveness of the proposed method and further demonstrate the broad potential of this technology for future applications, especially in fault diagnosis and state assessment based on signal features, providing fast and efficient solutions.

## Figures and Tables

**Table 1:** Lithium battery—four common faults.

Lithium Battery—Four Common Faults	
Type A	Normal (Type 1)
Type B	Over-discharging (Type 2)
Type C	Overcharging (Type 3)
Type D	Leakage (Type 4)
Type E	Aging (Type 5)

**Table 2:** Experimental equipment.

Equipment	Model	Specification
Programmable DC Power Supply	ITECH-IT6302	Output voltage: 0–30 V, Output current: 0–3 A, max power: 90 W
Programmable Electronic Load	ITECH-IT8512A	Max input voltage: 120 V, Max input current: 30 A, Max input power: 300 W
NI PXIe Chassis	NI PXI-5105	60 MHz sampling rate, 8 synchronized channels, and 12-bit resolution
Signal Generator	Agilent 33220A	Frequency range: 1 µHz to 20 MHz Modulation: AM, FM, PM, FSK Waveforms: sine, square, ramp, and pulse

**Table 3:** Results of lithium-ion battery fault detection based on CNN.

Fault Types	Training Pattern	Testing Pattern	Accuracy (%)
Type A	400	200	99.9
Type B	400	199	
Type C	400	200	
Type D	400	200	
Type E	400	200	

**Table 4:** Lithium-ion battery fault detection performance comparison.

Algorithm	Training Time (s)	Testing Time (s)	Epoch	Accuracy (%)
SDP + CNN	121.9	0.3970	50	95.7
SDP + Res-Net18	232.0	0.7095	50	89.8
SDP + VGG-19	369.6	0.0107	50	94.4
SDP + CNN	218.5	0.4457	100	99.9
SDP + Res-Net18	458.6	0.7602	100	91.5
SDP + VGG-19	723.5	0.0067	100	96.0

## Data Availability

Data are contained within the article.
